# A Theoretical Review on the Role of English as a Foreign Language Teachers’ Self-Disclosure in Shaping Classroom Climate and Immediacy

**DOI:** 10.3389/fpsyg.2022.945046

**Published:** 2022-07-01

**Authors:** Jing Qin

**Affiliations:** School of Foreign Languages, Xinxiang Medical University, Xinxiang, China

**Keywords:** EFL teacher, self-disclosure, classroom climate, interpersonal communication skills, immediacy

## Abstract

Teachers’ interpersonal communication skills and strategies have been widely considered effective pedagogical tools in academia. Despite the growing research in this area, unraveling the power of English as a foreign language (EFL) teachers’ self-disclosure in shaping classroom climate and immediacy has been relatively left intact. To fill the gap and provide new insights into this strand of research, the present article was an effort to present a theoretical analysis of the interplay of self-disclosure, classroom climate, and immediacy. In so doing, the definitions, conceptualizations, dimensions, underlying theories, and empirical evidence in support of the interaction among these three constructs were presented. Moreover, practical implications for EFL teachers, teacher trainers, and L2 scholars were provided to raise their awareness of interpersonal communication skills and their outcomes in academia. Finally, the study provided some suggestions for further research in this line of inquiry.

## Introduction

Teaching is by nature a communicative act and the way the teacher communicates with his/her pupils and maintains that interrelationship influences various aspects of education (Mazer, 2013; Hodge, 2014; Rajabi, 2015; Aghaei et al., 2020). An interpersonal relationship involving both teachers and students promotes teaching effectiveness and academic learning (Ballester, 2013; Sheybani, 2019). In English as a foreign language (EFL) contexts where the learners generally depend on their teachers, these are the teachers that are seen as the key to learning and teaching quality and communication (Pishghadam et al., 2021; Cui, 2022). They must be able to use different communication strategies to generate and sustain interest, participation, engagement, and interaction among students and establish a friendly environment for education (Habash, 2010; Xie and Derakhshan, 2021). One of the pivotal features of teacher-student interrelationship is the existence of self-disclosure on the part of both teachers and students (Safaei and Shahrokhi, 2019). As put by Jourard and Jaffe (1970), self-disclosure is, “the act of revealing personal information to others” (p. 2). It is an effective communicative and pedagogical tool in the classroom through which one verbally reveals his/her private feelings, attitudes, experiences, thoughts, and beliefs to another person (Chesebro, 2002; Vogel and Wester, 2003; Gkonou et al., 2018; Safaei and Shahrokhi, 2019).

In second/foreign language education, teacher self-disclosure (TSD, hereafter) refers to a teacher’s statements about self that may or may not be pertinent to course content, but reveal information about him/her that students are unlikely to obtain from other sources (Sorensen, 1989). The construct of TSD has been identified to produce numerous positive academic outcomes in L2 contexts from enhancing learners’ engagement, interest, and participation to teachers’ effectiveness and classroom rapport (Cayanus and Martin, 2016; Henry and Thorsen, 2021; Cui, 2022). Moreover, TSD facilitates the ground for the establishment of a constructive classroom climate as a significant factor in students’ psychological and educational growth (Grewe, 2017; Weber et al., 2021). Classroom climate is a dynamic and collective construct that is created by both individual and social perceptions of a classroom as a learning milieu (Eder, 2002; Wang and Degol, 2016). It has emanated from social psychology that perceives the classroom as a psychosocial and physical atmosphere for learning and teaching (Gedamu and Siyawik, 2015). Given its overarching essence, classroom climate can entail different classroom aspects including teachers’ and students’ characteristics, behaviors, and their interactions (Deng, 1992; Weber et al., 2021). Therefore, it can influence both parties’ academic performance and wellbeing in L2 contexts (Cohen et al., 2009; Eder, 2018). As research corroborates, classroom climate is a complex variable that is influenced by different personal, contextual, and cultural factors (Heitzmann, 2009). One of the most critical elements of an effective classroom climate is immediacy among students and the teacher (Gedamu and Siyawik, 2015). The term was originally proposed by Mehrabian (1969) as a communication behavior to elucidate the degree of proximity between people (Finn and Schrodt, 2012). It involves various verbal and non-verbal behaviors and strategies that teachers and students utilize in the class to form a sense of closeness and cohesiveness with one another (Dickinson, 2017; Delos Reyes and Torio, 2020). The existence and enhancement of immediacy in EFL classrooms have been found to bring about empowerment (Cakir, 2015), engagement (Marx et al., 2016), clarity and credibility (Zheng, 2021), attention (Bolkan et al., 2017), and reduces classroom anxiety (Kelly et al., 2015). However, the role of TSD, which is an effective strategy to establish classroom rapport and immediacy, has been widely ignored in the current literature. This gap is significant because the absence of a friendly academic context in which teachers feel free to reveal information about themselves may cause a distance between the teacher and students. This, in turn, leads to a rigid and non-productive learning atmosphere. Motivated by this shortcoming, this article aimed to cast some light on the possible impact of TSD on classroom climate and immediacy by reviewing the theoretical and empirical foundations of these constructs including their conceptualizations, definitions, dimensions, and lines of research. By doing so, the study provides promising insights about the power of TSD in facilitating L2 education in a friendly milieu.

## Background

### The Concept of Self-Disclosure

The concept of self-disclosure was originally studied in humanistic psychology, interpersonal relationships, and communication studies to refer to the act of revealing and sharing personal information with others (Jourard, 1971). The term entered into educational research and practice in the 1970s by Wheeless and Grotz (1976) who regarded self-disclosure as any message about the self that individual shares with other people. Theoretically, the concept of self-disclosure is supported by social penetration theory (SPT) which contends that people may share superficial, social, and/or intimate information to others (Jebbour, 2021). Superficial information comprises biographical information (e.g., names, birthplace, and hobbies), social information involves revealing beliefs, feelings, perspectives, experiences, attitudes, and so forth, and intimate information pertains to the act of communicating private secrets like relationships and family problems with others (Derlega et al., 2008; Cayanus and Martin, 2016; Jebbour, 2021). Likewise, Jourard (1971) argued that self-disclosure can include intimate topics (e.g., family concerns, financial status, and preferences) and non-intimate topics (e.g., political preference, personal hobbies, and interests). It plays a key role in forming close relationships (Derlega et al., 1993) and creating “comfort” as one of the representations of “social competence” (Wei et al., 2005, p. 602). It is essential to note that self-disclosure is a mutual construct in that its quality and content is determined not only by the discloser but also the context and the recipient (Farani and Fatemi, 2014). As pinpointed by Jourard (1971), self-disclosure generates self-disclosure, hence in language education this concept can create a sense of interpersonal bond in a counselor-client relationship that paves the way for many academic outcomes to emerge.

### Teacher Self-Disclosure

As teaching is a communicative process in which the teacher and students constantly negotiate meanings and send messages, the concept of self-disclosure is of paramount significance in this job (Simonds and Cooper, 2011; Shoeib, 2018). The quality of teachers’ communication skills also plays a crucial role in the pedagogical and learning processes that occur in the classroom (Jebbour and Mouaid, 2019). One of such skills is teacher self-disclosure (TSD) which refers to the act of revealing or sharing personal information, experiences, and sometimes close relationships with the students in order to clarify the content (Gkonou et al., 2018; Jebbour and Mouaid, 2019). Moreover, Bazarova (2012) defines TSD as teachers’ deliberate unveiling of personal and professional information and sharing it with students and colleagues in an attempt to build rapport and enhance the sense of intimacy. In a similar manner, Jebbour (2018) operationally defined TSD as teachers’ verbal communication of personal information as he/she is explaining the course content to learners in the class. To produce positive outcomes, TSD must be provided sufficiently and judiciously while it is relevant and meaningful for the students’ and the course content (Rajab, 2019).

### The Dimensions of Self-Disclosure

**FIGURE 1:**
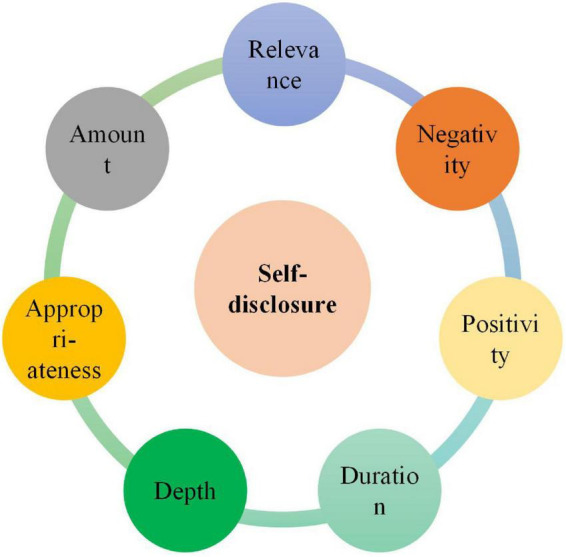
The dimensions of self-disclosure.

The concept of self-disclosure as a multi-dimensional construct has been claimed to be comprised of seven dimensions in the available literature (Cayanus and Martin, 2008; Jebbour, 2018). They include *amount*, *duration*, *depth, positivity, negativity, relevance*, and *appropriateness* (Figure 1). Amount refers to the degree to which a teacher employs self-disclosure in the class, while duration concerns the time that he/she spends sharing personal information or disclosing self to others. The next dimension is the depth which has to do with the degree of intimacy of one’s shared information (West and Turner, 2010). Furthermore, positivity pertains to the act of sharing or disclosing good or positive aspects of one’s life experiences with others, while negativity concerns the bad aspects of life (Jebbour, 2018; Wang and Guan, 2020). Relevance, as the name suggests, concerns sharing personal information and experiences with students that are related to the course content (Cayanus et al., 2009). Finally, appropriateness pertains to the degree of social acceptability of a self-disclosure revealed in the classroom. As pinpointed by Zhang et al. (2009) and Cayanus and Heisler (2013), sharing information about personal life experiences, family, friends, hobbies, and interests is appropriate, while those related to sex, religion, and politics are inappropriate to be disclosed in the classroom.

### The Purposes and Outcomes of Teacher Self-Disclosure in L2 Education

In L2 education, teachers usually take advantage of self-disclosure as an instructional tool to explain the course content, supplement the materials, stimulate students’ interests, gain students’ trust, and provide a living curriculum for learning and communication (Cayanus, 2004; Safaei and Shahrokhi, 2019). Moreover, TSD is intended to establish classroom interactions and interpersonal relationships with students, increase credibility, explain course content, and maintain students’ attention (Webb, 2014). If provided wisely in L2 classrooms, TSD can produce different positive academic outcomes such as students’ increased motivation, engagement, academic performance, content knowledge, interest, passion, social interactions, and enthusiasm to communicate in the classroom (Sorensen, 1989; Zhang et al., 2008; West and Turner, 2010; Cayanus and Martin, 2016; Wang and Guan, 2020; Liu and Zhu, 2021, among others). Moreover, TSD has been identified to benefit teachers as well. This communication strategy can establish a friendly rapport in the class, improve teaching effectiveness, and forms a relaxing climate for teaching and learning (Farani and Fatemi, 2014; Sanders, 2014; Henry and Thorsen, 2021). When both the teacher and students frequently but judiciously use self-disclosure to establish a sense of proximity, a democratic, friendly, and stress-free climate will be created that fosters many other favorable academic outcomes. This is because classroom climate operates as an umbrella that covers many aspects of education as explained in the following section.

### Classroom Climate: Definitions and Related Terms

Classroom climate refers to the overall learning environment, social climate, and the emotional and physical aspects of a class (Eder, 2018). It is a reflection of stakeholders’ opinions of their academic experience (Reid and Radhakrishnan, 2003). Moreover, it has been considered as a subjective and collective perception of a class, its features, members, and their interactions (Weber et al., 2021). To put it simply, classroom climate pertains to teachers’ and students’ prevailing mood and attitudes toward a classroom which can be positive or negative. A positive classroom climate is welcoming and supportive of education, while a negative classroom climate is destructive and harmful for both the teachers and students. Given its broad scope, the concept of classroom climate has not been given ample theoretical and operational definitions in L2 education. However, it has been differentiated from school climate and classroom culture.

School climate concerns the shared patterns of experience among all members of a school and reflects norms, values, objectives, interpersonal relationships, instructional behaviors and practices, and organizational structures (Thapa et al., 2013). Furthermore, classroom culture is a macro construct that affects almost all aspects of language learning (Altun, 2013). It pertains to the shared behaviors, beliefs, value systems, teaching and learning methods, relationships, and unwritten rules in a classroom (Cakiroglu et al., 2012). The demarcation between classroom culture and climate is that climate has to do with the social-ecological setting of learning that affects stakeholders’ attitudes, performance, perceptions, behaviors, self-concept, and wellbeing (Moos, 1979). In simple terms, climate is one’s general feeling about classroom relations, participation, and experience (Gabryś-Barker, 2016). On the contrary, classroom culture encompasses stakeholders’ shared values, traditions, patterns of belief and behavior, and relationships in the classroom (Cakiroglu et al., 2012). It is also regarded as a macro concept that changes over an extended period of time (Gruenert, 2008). Therefore, as pinpointed by Gruenert (2008), climate entails “attitudes and moods”, while culture entails “values and belief systems” of a classroom.

### Positive Classroom Climate

A positive classroom climate is believed to be established when both the teacher and students are engaged in interpersonal interactions, have classroom rapport, and co-construct the learning environment (Sidelinger and Booth-Butterfeld, 2010; Frisby et al., 2014). Research shows that a sustainable positive classroom climate can foster students’ motivation, satisfaction, learning, engagement, participation, wellbeing, and reduces their anxiety and apprehension (Graham and Gisi, 2000; Ellis, 2004; Norton, 2008; Barr, 2016). Such a positive environment is of paramount importance because when EFL teachers and students practice in a positive context, they can be more focused, relaxed, and eager to hit higher targets and be the best of who they can be. It is noteworthy that one of the most important elements of a positive classroom climate is the abundance of interpersonal communication skills. Among many interpersonal communications skills available in the literature, TSD and immediacy can play a quintessential role in establishing a learning atmosphere that connects teacher-students’ emotions to the instructional process. This is warranted in that a learning environment in which the teacher and students feel safe, accepted, and respected begets self-disclosure of personal information and a sense of proximity between the teacher and his/her students.

### The Concept and Main Theories of Immediacy

The concept of immediacy as an interpersonal communication strategy was coined by Mehrabian (1969) to refer to the degree of proximity between people. For Richmond (2002), immediacy is a perceived sense of physical and psychological closeness between individuals that creates interest and enthusiasm for interaction. In academia, the term immediacy concerns the use of various communication clues and behaviors to decrease the psychological/physical distance among stakeholders (Christophel and Gorham, 1995). They are impressive behaviors that transmit motivation, involvement, interest, and eloquence (Sheybani, 2019). To put it differently, immediacy is the use of verbal, non-verbal, and a combination of other expressive tools by teachers and students to constitute a positive rapport in the classroom (Ballester, 2013; Dickinson, 2017; Zheng, 2021).

As research signifies, there are two types of classroom immediacy, namely verbal and non-verbal immediacy. Verbal immediacy concerns verbal messages that demonstrate empathy, reward, kindness, willingness, inclusiveness, praise, openness, and humor in communication (Ballester, 2013). On the contrary, non-verbal immediacy refers to demeanors that intend to establish physical and emotional proximity in the classroom and increase students’ attention, participation, and course content liking (Richmond and McCroskey, 2000). Mostly, EFL teachers employ non-verbal immediacy clues to construct closeness, liking, and warmth that are transferable to students. As put by Stilwell (2018), non-verbal immediacy behaviors involve *proxemics* (distance), *haptics* (touch), vocalics (stress, pitch, tone, intonation, gesture, posture), *kinesics* (body movement/orientation), *oculesics* (eye contact), *classroom environment* (e.g., seating arrangements), and *chronemics* (time).

Concerning theoretical underpinnings, three theories underpin classroom immediacy in education. They include Bowlby’s (1969) attachment theory (AT), Maslow’s (1962) hierarchy of needs theory and loving pedagogy (Barcelos, 2020; Wang et al., 2022). AT is the foundation of developmental psychology in that it explicates the interpersonal patterns among people. This theory posits that an individual’s attachment to others shapes a behavior in him/her that can become self-directed in the future. In the context of language education, AT highlights emotional connections among teachers and students in establishing rapport, practices, experiences, and engagement in classroom activities (Riley, 2011; Zheng, 2021). Based on AT, students with emotional attachment to their teacher are more relaxed, socialized, motivated, engaged, and risk-takers (Bergin and Bergin, 2009). This sense of attachment provides a secure learning environment for students to grow and gain academic expertise.

**FIGURE 2:**
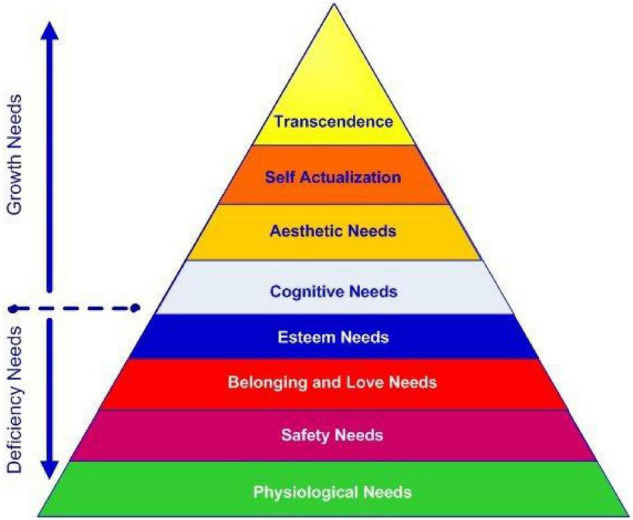
The hierarchy of needs theory (Maslow, 1962).

The second related theory is the hierarchy of needs theory proposed by Maslow (1962) which was a breakthrough at its time (Figure 2). This theory maintains that a person’s basic human needs must be satisfied before he/she reaches ultimate performance (Freitas and Leonard, 2011). The theory is in the form of a pyramid in which the fulfillment of basic needs enhances one’s motivation and enthusiasm to try harder (Maslow, 1962).

Undoubtedly, all these human needs are vital in one’s life and education, yet the psychological needs (belongingness and intimacy in communities) are more powerful in educational contexts in that they can pay off the other deficiencies. Because of this, good teachers usually form strong emotional bonds with their students by using different immediacy clues and behaviors to manifest their concern for students’ psychological needs (Teven and Hanson, 2004). The third theory related to this study is “loving pedagogy” which has recently gained momentum in educational research (Loreman, 2011; Wang et al., 2021, 2022). This theory highlights the importance of teachers’ care, sensitivity, and empathy toward their students’ needs, learning, and development (Yin et al., 2019). It has been found that adopting a pedagogy of love can enhance students’ self-esteem, autonomy, motivation, critical-thinking, positive interpersonal behaviors, and academic achievement (Xie and Derakhshan, 2021). Loving pedagogy has also been identified to affect teaching and construct teachers’ professional identity (Barcelos and Coelho, 2016; Barcelos, 2020).

### Related Studies

Different empirical studies have been conducted on the variables reviewed in this article. Concerning self-disclosure, a growing body of research indicates that it increases EFL students’ classroom engagement, participation, attention, motivation, achievement, willingness to communicate, interpersonal skills, passion, social competence, speaking skills, writing skills, and decreases their anxiety (Pishghadam and Torghabeh, 2009; Hosseini and Tabatabaee, 2010; Serag, 2011; Cayanus and Martin, 2016; Henry and Thorsen, 2021; Liu and Zhu, 2021). Moreover, TSD has been found to have a strong correlation with teachers’ teaching style, effectiveness, clarity, assertiveness, and responsiveness (Cayanus and Martin, 2004; Rajab, 2019; Safaei and Shahrokhi, 2019). Another area that TSD can influence is classroom climate (Farani and Fatemi, 2014). Scientific evidence confirms that classroom climate, especially positive classroom climate is correlated with many academic outcomes including improved interpersonal communication, classroom rapport, learning motivation, job satisfaction, classroom involvement, wellbeing, and decreased anxiety (Graham and Gisi, 2000; Ellis, 2004; Norton, 2008; Sidelinger and Booth-Butterfeld, 2010; Frisby et al., 2014; Barr, 2016).

Moreover, immediacy as one of the most outstanding consequences of TSD and positive classroom climate has been found to improve students’ motivation, attention, involvement, WTC, interpersonal communication skills, psychological empowerment, clarity, and credibility (Cakir, 2015; Marx et al., 2016; Bolkan et al., 2017; Sheybani, 2019; Zheng, 2021). Although previous studies done on these three variables have provided insightful ideas, they have mostly been one-shot, associational studies without considering their situated nature and linkage with interpersonal communication skills. Moreover, scrutinizing the interplay of TSD, classroom climate, and immediacy, at the same time, has not captured enough scholarly attention, to date. More specifically, the way each of these variables influences, determines and predicts the other ones in EFL contexts is yet under-researched. Hence, future studies are demanded to cast more light on the interaction of TSD, classroom climate, and immediacy.

## Concluding Remarks

In this review article, it was pinpointed that TSD is a facilitator and predictor of EFL classroom climate and immediacy, which in turn, generate several positive academic outcomes of both teachers and students. It was also claimed that when EFL teachers provide more self-disclosure to their students, a strong sense of rapport would be created in the class that installs a positive classroom climate for education that is laden with immediacy cues. Based on these, it is contended that the present theoretical review would have valuable contributions to the field by acting as a response to the necessity and significance of including awareness-raising of interpersonal communication skills and their outcomes in academia. It can also be beneficial for EFL teachers in that they can understand the power of interpersonal communication strategies like self-disclosure in shaping the overall classroom climate and a sense of proximity in the classroom. They can also use verbal and non-verbal immediacy cues to reinforce classroom rapport and academic performance of the students. Furthermore, this study can be helpful for teacher trainers by showing them the criticality of interpersonal communication skills in many aspects of L2 education. Hence, they can offer training programs like workshops and seminars on different communication skills and practical ways of applying them in the classroom. Finally, L2 researchers may find this review fruitful in that they can carry out further studies on this line of research and bridge the existing gaps. More particularly, they can conduct mixed-methods, qualitative, and longitudinal studies on TSD, classroom climate, and immediacy to provide richer data as opposed to one-shot, correlational studies common in this area. Likewise, given the culture and context specificity of these three constructs, they are recommended to run cross-cultural studies to unpack the role of cultural factors in the interaction of the reviewed constructs. Additionally, future researchers are suggested to focus on the situated nature of the interplay among TSD, classroom climate, and immediacy considering the role of more variables, socio-economic factors, and power-related factors embedded in the context. Finally, the relationship between TSD and other interpersonal communication skills such as clarity, confirmation, credibility, stroke, care, etc. is also an interesting topic for future research.

## Ethics Statement

The studies involving human participants were reviewed and approved by the Xinxiang Medical University Academic Ethics Committee. The patients/participants provided their written informed consent to participate in this study.

## Author Contributions

The author confirms being the sole contributor of this work and has approved it for publication.

## Conflict of Interest

The author declares that the research was conducted in the absence of any commercial or financial relationships that could be construed as a potential conflict of interest.

## Publisher’s Note

All claims expressed in this article are solely those of the authors and do not necessarily represent those of their affiliated organizations, or those of the publisher, the editors and the reviewers. Any product that may be evaluated in this article, or claim that may be made by its manufacturer, is not guaranteed or endorsed by the publisher.
